# Transgenic inhibition of astroglial NF-κB restrains the neuroinflammatory and neurodegenerative outcomes of experimental mouse glaucoma

**DOI:** 10.1186/s12974-020-01930-1

**Published:** 2020-08-28

**Authors:** Xiangjun Yang, Qun Zeng, Mine Barış, Gülgün Tezel

**Affiliations:** Department of Ophthalmology, Vagelos College of Physicians and Surgeons, Columbia University, Edward S. Harkness Eye Institute, 635 West 165th Street, New York, NY 10032 USA

**Keywords:** Astroglia, Glaucoma, IκKβ, Neurodegeneration, Neuroinflammation, NF-κB

## Abstract

**Background:**

Glia-driven neuroinflammation promotes neuron injury in glaucoma that is a chronic neurodegenerative disease of the optic nerve and a leading cause of irreversible blindness. Although therapeutic modulation of neuroinflammation is increasingly viewed as a logical strategy to avoid inflammatory neurotoxicity in glaucoma, current understanding of the molecular regulation of neuroinflammation is incomplete, and the molecular targets for immunomodulation remains unknown. Growing datasets pointed to nuclear factor-kappaB (NF-κB), a key transcriptional activator of inflammation, which was identified to be most affected in glaucomatous astroglia. Using a cell type-specific experimental approach, this study aimed to determine the value of astroglial NF-κB as a potential treatment target for immunomodulation in experimental mouse glaucoma.

**Methods:**

Neuroinflammatory and neurodegenerative outcomes of experimental glaucoma were comparatively analyzed in mice with or without cre/lox-based conditional deletion of astroglial *IκKβ*, which is the main activating kinase involved in IκB degradation through the canonical pathway of NF-κB activation. Glial responses and the inflammatory status of the retina and optic nerve were analyzed by cell morphology and cytokine profiling, and neuron structure and function were analyzed by counting retinal ganglion cell (RGC) axons and somas and recording pattern electroretinography (PERG) responses.

**Results:**

Analysis of glial inflammatory responses showed immunomodulatory outcomes of the conditional transgenic deletion of *IκKβ* in astroglia. Various pro-inflammatory cytokines known to be transcriptional targets for NF-κB exhibited decreased production in *IκKβ*-deleted astroglia, which included TNF-α that can induce RGC apoptosis and axon degeneration during glaucomatous neurodegeneration. Indeed, transgenic modulation of inflammatory responses by astroglial *IκKβ* deletion reduced neurodegeneration at different neuronal compartments, including both RGC axons and somas, and protected PERG responses.

**Conclusions:**

The findings of this study support a key role for astroglial NF-κB in neuroinflammatory and neurodegenerative outcomes of experimental glaucoma and the potential of this transcriptional regulator pathway as a glial treatment target to provide neuroprotection through immunomodulation. By pointing to a potential treatment strategy targeting the astroglia, these experimental findings are promising for future clinical translation through transgenic applications to improve the treatment of this blinding disease.

## Background

Glaucoma, a leading cause of irreversible blindness, is characterized by chronic degeneration of retinal ganglion cell (RGC) axons in the optic nerve, death of RGC somas in the retina, and loss of synapses in the retina and brain. Although this blinding disease affects approximately 80 million people worldwide, no effective treatment is available to prevent progressive neuron injury and vision loss. Among multiple pathogenic mechanisms implicated in glaucoma, neuroinflammation, relying on reactive glia, is increasingly recognized to play an important role [1–4]. Despite subtype-specific, topographic, and temporal variations, glial cells profoundly respond to glaucoma-related tissue stress/injury by morphological, molecular, and functional alterations. Besides innate immunity, prolonged responses of these resident immunocompetent cells may also stimulate adaptive immunity, and autoreactive T cells, autoantibodies, and complement components may become potent stimuli to harm RGCs during glaucomatous neurodegeneration [1–4]. While growing information points to independent molecular processes for glaucomatous degeneration of RGCs at different subcellular regions [5], glia-driven neuroinflammation appears to promote neuron injury throughout the visual pathway. Viewing the big picture of glaucoma and respecting the diverse roles of glia (which are normally neurosupportive versus neurodestructive under glaucoma-related stress), these critical cells constitute a promising treatment target for the modulation of neurodegenerative inflammation at different injury sites from the retina to the brain.

Multiple subtypes of glia, including both macroglia and microglia, are linked to glaucomatous neurodegeneration [1–4]. Macroglia consist of glial fibrillary acidic protein (GFAP)-expressing astrocytes with the addition of Müller glia in the retina, non-GFAP-expressing lamina cribrosa cells in the optic nerve head, and oligodendrocytes in the optic nerve. This study was focused on “GFAP-expressing astroglia,” because astroglial responses are rapidly produced, broadly manifested, and robustly persisting during the chronic disease period with widespread impacts in glaucoma. As opposed to more transient and restricted responses of microglia, astroglia appear to be excellent candidates for a potential treatment target to restore immune homeostasis and improve neuron survival.

Improved molecular understanding is key toward developing a therapeutic approach through immunomodulation. However, despite a growing interest in the inflammatory mechanisms of neurodegeneration in glaucoma, the current understanding of molecular regulation is incomplete, thereby precluding new treatments. Based on recent molecular profiling and histopathological analysis of human donor eyes with glaucoma [6] and animal models [7], astroglial nuclear factor-kappaB (NF-κB) seems to be critical for the transcriptional regulation of neuroinflammation that involves cytokine signaling, toll-like receptor (TLR) signaling, and inflammasome activation [6–8]. Evidently, the pro-inflammatory and neurotoxic mediators produced through NF-κB-regulated inflammation signaling can exacerbate neuron injury [9, 10]. Although inflammatory outcomes of NF-κB activation have been studied in non-glaucomatous injury models of the optic nerve [11–13], the pathogenic importance of astroglial NF-κB in glaucoma remains unclear. This study aimed to determine the importance of astroglial NF-κB for neuroinflammation in glaucoma and to value its potential as an immunomodulation target for neuroprotection.

Transcriptional activity of NF-κB depends on the degradation of a specific inhibitory protein, inhibitor of kappaB kinase (IκB), via a process that requires specific kinases (IκB kinase, IκK) for phosphorylation and polyubiquitination. The IκK-subunit β (IκKβ), but not IκKα or IκKγ (the regulatory subunit), is the main activating kinase [14] involved in IκB degradation through the canonical pathway of NF-κB signaling with inflammatory outcomes [15–17]. Given the tight regulation of NF-κB by IκKβ (which activates its DNA binding), IκKβ has been an efficient treatment target to modulate NF-κB activation [18, 19]. In order to dissect the role of astroglial NF-κB, we utilized the cre-recombinase (cre)/loxP system under the regulation of the mouse glial GFAP promoter to knock-out the transcription of *IκKβ* in astroglia. We then analyzed the transgenic effects on neuroinflammatory and neurodegenerative outcomes of experimentally induced glaucoma. Glial inflammatory responses were analyzed by cell morphology and cytokine profiling, and the neuron structure and function were analyzed by counting neurons and recording pattern electroretinography (PERG) responses. The findings of these analyses supported a key role for NF-κB in astroglia-driven neurodegenerative inflammation in experimental glaucoma, thereby pointing to astroglial NF-κB as a favorable treatment target to provide immunomodulation for neuroprotection in glaucoma.

## Methods

### Mice

This study included 2- to 3-month-old mice that were housed in 12-h light/dark cycle and received food and water ad libitum. The utilized transgenic mouse line was generated by breeding the *GFAP*-*cre/ERT2* [20] (stock no. 012849; The Jackson Laboratory, Bar Harbor, ME) into IκKβ^f/f^ mice that were generated and provided by Dr. Michael Karin at UCSD [21, 22], both of which were on a C57BL/6J background, followed by crossing of the offsprings with IκKβ^f/f^. The IκKβ^f/f^ mice that were generated by flanking exon 3, which codes for the IκKβ activation loop, with 2 *lox*P sites, have been useful in previous studies of immune cells or brain neurons. For conditional recombination, tamoxifen (dissolved in corn oil) was given to crossbreds by intraperitoneal injection (5 mg/40 g mouse) once a day for 5 consecutive days. Controls including IκKβ^f/f^ mice wild-type for *cre* (and C57BL/6J wild-type for the floxed allele with/without *cre*) received similar tamoxifen injections. Additional controls included the littermates given oil vehicle only. Tamoxifen was injected at the time of microbead injection for experimental induction of ocular hypertension. A PCR-based method with primers detecting *cre* (5′-GCC AGT CTA GCC CAC TCC TT-3′, and 5′-TCC CTG AAC ATG TCC ATC AG-3′) and *IκKβ*^*flox*^ (5′-CCT TGT CCT ATA GAA GCA CAA C-3′, 5′-GTC ATT TCC ACA GCC CTG TGA-3′) alleles was used to verify the genotype in cre-loxP mice.

### Microbead occlusion to model chronic ocular hypertension-induced glaucoma in mouse

Intraocular pressure elevation was induced by anterior chamber microbead injection [23] by applying a modification [24] for more consistent ocular hypertension and greater axonal loss. Similar to our previous studies [25], 4 μl of polystyrene microbeads (a mixture of 6 μm and 1 μm beads) followed by 1 μl viscoelastic (10 mg/ml sodium hyaluronate to push the beads into the anterior chamber angle and prevent the bead reflux after injection cannula is removed) was injected into the anterior chamber of one eye using a Hamilton syringe (Hamilton Company, Reno, NV). The injection was repeated at week 4, because a single injection results in 4 to 6 weeks of ocular hypertension, but a second injection maintains the high intraocular pressure for a longer period of up to 12 weeks, resulting in approximately 50% axon loss [25, 26]. This commonly used microbead occlusion model with moderate IOP elevation for a relatively longer experimental period allowed us to model ocular hypertension-induced glaucoma in transgenic and control mice. This experimental model was also considered advantageous to study glia-driven neuroinflammation, because glial inflammatory responses develop as a consequence of ocular hypertension-related tissue stress/injury [25], while another commonly utilized glaucoma model, the hereditary glaucoma in DBA/2J mice, presents pre-existing inflammation.

Fellow eyes of mice were injected with an equivalent volume of physiologic saline. We measured intraocular pressure before and after injections and twice weekly thereafter with a TonoLab rebound tonometer (TioLat, Helsinki, Finland) under isoflurane anesthesia and calculated the intraocular pressure-time integral. The mice with an intraocular pressure increase above 40 mmHg were excluded from the study.

### PERG

To assess the functional status of RGCs in mice, PERG responses were recorded using a dual PERG system (Jörvec, Miami, FL) by following the protocols described by Dr. Porciatti [27, 28]. Briefly, mice were anaesthetized using ketamine and xylazine, and the body temperature was maintained at 37 °C on a feedback-controlled heated stage that was monitored by a rectal thermometer. Balanced salt solution drops were applied to prevent corneal drying and maintain its transparency. The patterned stimuli (black-white reversing gratings of 0.05 cycles/degree, and 100% contrast) generated on LED panels were presented at each eye separately with slight different frequencies around 1 Hz. Right and left eye responses were extracted real-time using a single snout electrode. The waveforms acquired were retrieved using an asynchronous averaging method (including at least 1800 responses per eye). The PERG amplitude was evaluated peak-to-trough. The light-adapted flash ERG response (that did not change with ocular hypertension) was used as a control on all mice. All electrophysiological recordings were performed in a masked fashion.

### Optic nerve axon counting

One-μm-thick cross-sections of the optic nerve embedded in epoxy resin were used for imaging-based axon quantification in a masked fashion similar to our previous studies [25]. Images of the toluidine blue-stained sections were collected as non-overlapping tile images using Zeiss AxioObserver.Z1 microscope for wide-field fluorescence microscopy (Carl Zeiss, Thornwood, NY) and the Zen software (Blue Edition; Carl Zeiss) to allow axon counts representing the entire surface area of cross-sections as previously described [25]. Axon counting was performed by a researcher masked for the experimental group. After image acquisition, nerve outlines were manually traced on mosaics of images. Image processing determined the size and shape parameters to exclude intervening glia, myelin debris, and highly degenerated axons. The axon loss was determined by the ratio of axon counts in ocular hypertensive eye to normotensive fellow eye.

### RGC counting

RGCs were counted in retinal whole mounts after immunolabeling with an antibody to a marker protein, RNA-binding protein with multiple splicing, RPBMS (1:200; GeneTex, Irvine, CA; catalog number: GTX118619). Images were acquired on a laser scanning confocal microscope (Red A1, Nikon Ti Eclipse; Nikon, Melville, NY) using the NIS-elements AR5 software (Nikon). Non-overlapping tile images of the whole mounted retina were collected for a depth of 0–30 μm as z stacks (with 5 μm step size) at a magnification of 10. The images that were collapsed into two-dimensional images to generate maximum intensity projections were used for RGC counting similar to described for axon counting. As we previously described [25], besides RBPMS immunolabeling, an additional criterion included a minimal soma size of 10 μm to eliminate dying or phagocytized RGCs. The RGC loss was expressed as the ratio of RGC counts in ocular hypertensive to fellow eye. The researcher counting RGC numbers was blinded to the experimental group of samples.

### Analysis of glial morphology in the retina

Astroglial response was analyzed in confocal images of retinal whole mounts after GFAP immunolabeling. Images containing the projections made from the same number of image stacks were used to analyze astroglial morphology in a blinded (to experimental parameters) fashion. After initial hand-counting of GFAP+ cells, the intensity of GFAP immunolabeling was evaluated as the mean pixel intensity across each image (expressed as the mean GFAP intensity). In addition, in order to determine astroglial hypertrophy, the number of GFAP+ pixels was divided by the total number of pixels in each image (expressed as the percentage of GFAP coverage). The same imaging parameters were also analyzed for Iba1 immunolabeling of microglia. The values obtained from ocular hypertensive eyes were corrected to the values from normotensive controls and expressed as fold change in GFAP or Iba1 intensity and coverage.

### Tissue immunolabeling

Whole mounts of the retina, and 6-μm-thick histological sections of the paraffin-embedded retina and optic nerve tissues, were immunolabeled with specific antibodies, as previously described [6, 7, 25]. Briefly, the retinas dissected from enucleated eyes were fixed in 4% paraformaldehyde for 1 h at room temperature. Subsequently, retinas, and deparaffinized tissue slides, were rinsed with PBS and incubated in a blocking solution containing 1% BSA (Sigma-Aldrich; St. Louis, MO) and 0.3% Triton X-100 (ThermoFisher) in PBS for 1 h. This step was followed by incubation with primary antibodies in blocking solution at 4 °C overnight. After rinsing in PBS, retinal whole mounts and tissue slides were incubated with secondary antibodies in blocking solution for 1 h at room temperature. Rinsed retinas were then mounted on glass slides, and all slides were coverslipped with 50 μl of Fluoroshield mounting medium with 4′,6-diamidino-2-phenylindole dihydrochloride (DAPI; Abcam, catalog number: ab104139).

Primary antibodies used for immunofluorescence labeling included monoclonal antibodies to GFAP (1:300; Abcam, Cambridge, MA; catalog number: ab68428 and ab10062), Iba1 (1:300; Abcam, catalog number: ab178847), TNF-α (1:200; Abcam, catalog number: ab109322), or cre-recombinase (1:500; Sigma-Aldrich, catalog number: MAB3120). In addition, a monoclonal antibody to IκKβ (1:300; ThermoFisher, Waltham, MA; catalog number: MA5-16154) and a phosphorylation site-specific primary antibody to NF-κB subunit, p65 [phospho-Ser536] (1:500; Abcam, catalog number: ab86299) were used to study NF-κB activation pathway. A mixture of Alexa Fluor dye (488, 555, or 647)-labeled secondary antibodies (1:1000; ThermoFisher) was used for the secondary antibody incubation. For negative control, the primary antibody was replaced with serum or an inappropriate secondary antibody was used to determine species specificity. Immunolabeled whole mounts, and histological tissue sections, were imaged using the laser scanning confocal microscope (Red A1, Nikon Ti Eclipse; Nikon).

### Quantitative Western blot analysis of astroglia

Enriched samples of the retina and optic nerve (including the optic nerve head) astroglia were obtained by two-step immunomagnetic cell selection from dissociated cell mixtures using a similar methodology as we previously described [7]. In addition to papain-based enzymatic digestion and mechanical dissociation, optic nerve samples were subjected to myelin removal using specific magnetic beads (Miltenyi Biotec). After depletion of macrophage/microglia in the first step of immunomagnetic cell selection using a monoclonal antibody to CD-11b (1:10; Abcam, catalog number: ab8878), astroglia were selected from cell mixtures by incubation with magnetic beads bound to a monoclonal antibody to astrocyte cell surface antigen (ACSA)-2 (1:10; Miltenyl Biotech, catalog number: 130-099-138). Alternatively, a monoclonal antibody to ACSA-1 (GLAST) was used to select retinal Müller glia (1:10; Miltenyl Biotech, catalog number: 130-095-822). Our updated technique (with > 80% cell yields) isolates ~ 10 μg astroglia protein from 4 mouse retinas or optic nerves. The protein samples used in this study were pooled from ~ 40 eyes per group matched for the cumulative IOP exposure.

Protein extraction and immunoblotting followed a protocol similar to our previous studies [6, 7, 25]. For multiplex fluorescent Western blotting, immunoblot membranes were probed overnight with a phosphorylation site-specific primary antibody to NF-κB subunit, p65 [phospho-Ser536] (1:500; Abcam, catalog number: ab86299), or monoclonal antibodies to astroglia or neuron markers (GFAP; neuronal nuclei protein, NeuN; or neurofilament protein, NFP; 1:500; Abcam, catalog number: ab10062, ab177487, ab236122, respectively). Besides using stain-free gels, a β-actin antibody (1:1000; ThermoFisher, catalog number: MA5-15739, and Abcam, catalog number: ab179467) was mixed with the primary antibody for loading and transferring controls. The primary antibody incubation was followed by incubation with infrared dye-labeled secondary antibodies (1:10,000; Li-Cor, Lincoln, NE) for 1 h. Proteins were visualized and quantified using Odyssey Infrared Imaging system (Li-Cor), and the band intensity values were normalized to β-actin before calculating the glaucoma-related fold change in protein expression.

### Enzyme-linked immunosorbent assay

Protein lysates were obtained from retina and optic nerve (including the optic nerve head and the optic nerve segment proximal to the globe) samples and the isolated samples of glia by homogenization in a lysis buffer (50 mM HEPES-KOH pH 8.0, 100 mM KCl, 2 mM EDTA, 0.10% NP-40, 2 mM dithiothreitol, 10% glycerol, supplemented with protease and phosphatase inhibitors) as previously described [6, 7, 25]. Protein concentration was determined by a colorimetric analysis based on the Bradford protein assay (BioRad, Hercules, CA). A Multi-Analyte ELISArray kit (QIAGEN, Valencia, CA) was used for simultaneous profiling of 12 cytokines/chemokines (detection sensitivity at low pg/ml levels) as previously described [25]. All analyses were conducted in triplicated wells and included negative controls, and the positive and nonspecific binding controls provided by the kits. Concentrations were calculated from a standard curve, and the values were corrected for protein concentration.

### Statistical analysis

In order to minimize the influence of IOP variability among animals, the data collected from transgenic versus control mice were statistically analyzed (in a masked fashion) in groups matched for the intraocular pressure-time integral (calculated by first integrating the mean intraocular pressure over time in ocular hypertensive eye, then subtracting the intraocular pressure-time integral from that in normotensive fellow eye). The analyzed eyes had a cumulative IOP exposure between 200 and 400 mm Hg-days (corresponding to up to 50% neuron loss). For all experimental conditions, data were analyzed using a software (SigmaPlot, version 12.5; Systat Software, Inc., San Jose, CA). Experiments comparing differences across groups were analyzed using the one-way analysis of variance (ANOVA) followed by the pairwise multiple comparison test of Holm-Sidak, or the Kruskal-Wallis one-way ANOVA on ranks followed by the Tukey test for pairwise multiple comparisons. Statistical significance was considered for *P* values of less than 0.05. All data are presented as mean ± SD.

## Results

### Experimental modeling of ocular hypertension-induced glaucoma in mice with astroglia-targeted deletion of *IκKβ*

**Fig. 1:**
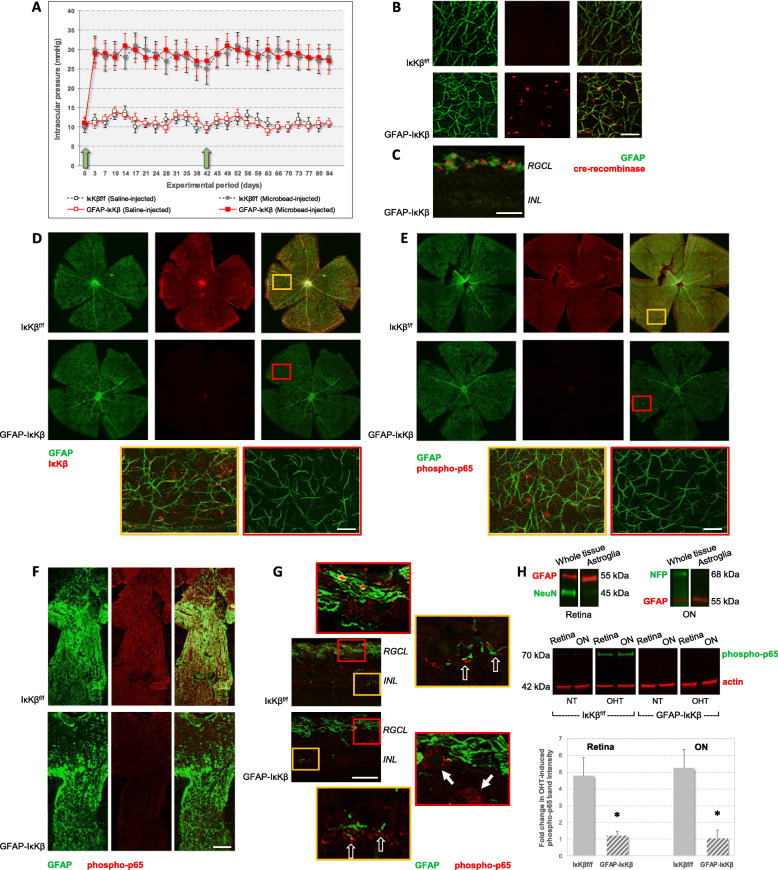
Experimental modeling of ocular hypertension-induced glaucoma in GFAP-IκKβ mice and controls. **a** Intraocular pressure elevation was induced in GFAP-IκKβ mice and transgenic controls (IκKβ^f/f^ mice wild-type for *cre*) by microbead injections into the anterior chamber of one eye. Fellow eyes received similar injections of physiological saline. Intraocular pressure curves were obtained over the experimental period of 12 weeks. Blue arrows show the time points for microbead (or saline) injections. Microbead injections resulted in a significant increase in intraocular pressure (***P < 0.001) with no significant difference between GFAP-IκKβ mice and IκKβ^f/f^ controls (*P* = 0.45). Immunolabeling of retinal whole mounts (**b**) and a histological section (**c**; RGCL, retinal ganglion cell layer; INL, inner nuclear layer) demonstrate cre-recombinase (red) expression in GFAP+ astroglia (green) after tamoxifen-injection in GFAP-IκKβ mice (not in IκKβ^f/f^ controls). In order to confirm astroglia-targeted deletion of *IκKβ*, we also analyzed IκKβ expression and downstream activity (by analysis of p65 phosphorylation) in retina and optic nerve samples obtained from transgenic mice and controls. Panels **d** and **e** show IκKβ, or phospho-p65 (red), immunolabeling of GFAP+ astroglia (green) in retinal whole mounts obtained from ocular hypertensive eyes of GFAP-IκKβ mice or IκKβ^f/f^ controls. Despite prominent localization of IκKβ (and phospho-p65) immunolabeling to GFAP+ astroglia in control mice, astroglial IκKβ (and phospho-p65) labeling was decreased in GFAP-IκKβ mice (scale bar, 100 μm). Yellow and red boxed areas on merged images in panels **d** and **e** are shown in higher magnification. Histological sections of the optic nerve (panel **f**) and retina (panel **g**) similarly showed decreased immunolabeling of GFAP+ astroglia for phospho-p65 (and decreased colocalization of GFAP and phospho-p65 immunolabeling) in ocular hypertensive GFAP-IκKβ mice relative to ocular hypertensive IκKβ^f/f^ controls (scale bar, 100 μm). Red or yellow boxed areas in panel **g** are shown in higher magnification. Note that although no phospho-p65 immunolabeling was detectable in GFAP+ astroglia in the RGC layer (corresponding to astrocytes), GFAP− neurons (white arrows) exhibited phospho-p65 immunolabeling in the GFAP-IκKβ retina. The GFAP+ cells in the inner nuclear layer (corresponding to Müller glia; translucent arrows) also immunolabeled for phospho-p65. As presented in panel **h**, for testing p65 phosphorylation by quantitative Western blot analysis, astroglia were isolated from retina and optic nerve (ON, including the optic nerve head) samples by immunomagnetic cell selection. Immunoblots of enriched astroglia proteins exhibited immunoreactivity for GFAP (red), an astroglia marker, but were negative for neuron markers (NeuN or NFP, green). When the immunoblots of astroglia proteins were probed with a phosphorylation site-specific antibody to NF-κB subunit p65 (green), band intensities (normalized to β-actin bands, red) indicated over four-fold increased expression of phospho-p65 with ocular hypertension (OHT) in IκKβ^f/f^ control mice (*P = 0.005, and *P* = 0.004 for retina and optic nerve samples, ON, respectively). However, both the basal expression in normotensive (NT) eyes and the ocular hypertension-induced upregulation of phospho-p65 were not detectable in the retina or optic nerve astroglia obtained from GFAP-IκKβ mice. Presented immunoblots and the quantitative data (mean ± SD) represent a minimum of 10 mice per group in triplicated analyses

For modeling of glaucoma in transgenic and control mice, intraocular pressure elevation was experimentally induced by anterior chamber microbead injections in one eye, and the physiological saline-injected fellow eye served as normotensive control. Microbead injections that were repeated at week 4 of the 12-week experimental period resulted in a significant increase in intraocular pressure relative to saline-injected control eyes (*P* < 0.001). Similar to transgenic controls **(**IκKβ^f/f^ mice wild-type for *cre*), microbead injections induced moderate elevation of intraocular pressure in GFAP-IκKβ mice. Figure 1a shows the intraocular pressure curve in microbead-injected or saline-injected eyes of GFAP-IκKβ mice and IκKβ^f/f^ controls. While the saline-injected eyes had a steady level of intraocular pressure that was maintained at average values below 12 mmHg through the experimental period of 12 weeks, microbead-injected eyes of GFAP-IκKβ mice or controls similarly exhibited a course of ocular hypertension (mean ± SD, 28.09 ± 3.98, and 27.86 ± 4.25 mmHg, respectively). No significant difference was detectable between the intraocular pressure elevation in GFAP-IκKβ mice or IκKβ^f/f^ controls (*P* = 0.45). Induction of intraocular pressure elevation (and the magnitude of neuron loss) was similar to previous studies of the same experimental model [25, 26].

In order to confirm astroglia-targeted deletion of *IκKβ*, we comparatively analyzed IκKβ expression and downstream activity (by analysis of p65 phosphorylation, a critical component of the canonical pathway of NF-κB activation) in the retina and optic nerve samples obtained from transgenic mice and controls with or without experimentally induced ocular hypertension. Figure 1b presents retinal whole mounts to exemplify cre-recombinase expression in GFAP+ astroglia after tamoxifen injection in GFAP-IκKβ and IκKβ^f/f^ mice. As illustrated in a histological section of the retina from a tamoxifen-injected GFAP-IκKβ mouse (in Fig. 1c), the GFAP+ cells in the RGC layer (corresponding to astrocytes) exhibited prominent immunolabeling for cre-recombinase. As shown in Fig. 1d, immunolabeling of retinal whole mounts demonstrated a prominent drop in the ocular hypertension-induced IκKβ labeling of GFAP+ astroglia in GFAP-IκKβ mice compared to IκKβ^f/f^ controls. In addition, retinal whole mounts (Fig. 1e) and histological sections of the optic nerve (Fig. 1f) obtained from ocular hypertensive IκKβ^f/f^ controls exhibited astroglial immunolabeling for phospho-p65. However, astroglial expression of phospho-p65 was undetectable by immunolabeling of retinal whole mounts or optic nerve sections from GFAP-IκKβ mice. In order to better illustrate the immunolabeling in different retinal layers, Fig. 1g presents histological sections of the retina from ocular hypertensive GFAP-IκKβ and IκKβ^f/f^ mice. Note that similar to optic nerve, phospho-p65 immunolabeling of the GFAP+ astroglia in the RGC layer (corresponding astrocytes) was not detectable in the ocular hypertensive GFAP-IκKβ retina. Yet, the GFAP+ cells in the inner nuclear layer (corresponding to Müller glia) exhibited phospho-p65 immunolabeling in both of these ocular hypertensive GFAP-IκKβ and IκKβ^f/f^ retinas.

We also tested p65 phosphorylation by quantitative Western blot analysis of enriched astroglia proteins that were obtained from retina and optic nerve samples by immunomagnetic cell selection. Our previous work verified the isolated samples of astroglia by RT-PCR [29] and immunoblotting for specific markers and ensured the feasibility of their analysis in ocular hypertensive mouse eyes [7]. Immunoblots verified the enrichment of astroglia herein as well (Fig. 1h). Western blot analysis of enriched samples of astroglia used a phosphorylation site-specific antibody to NF-κB subunit p65. The phosphorylation site was Ser536 that is the phosphorylation site by IκKβ for stimulation of transcriptional activity and nuclear import [30, 31]. As also shown in Fig. 1h, band intensities of retina and optic nerve astroglia proteins for phospho-p65 exhibited an over four-fold increase in ocular hypertensive versus normotensive *IκKβ*^*f/f*^ mice (*P* = 0.005 and *P* = 0.004 for retina and optic nerve samples, respectively). However, when we similarly analyzed the astroglia proteins obtained from GFAP-IκKβ mice, both the basal expression and ocular hypertension-induced upregulation of phospho-p65 were not detectable.

Thus, similar to human glaucoma [6] and experimental rat glaucoma [7], astroglial NF-κB was activated within 12-weeks of moderate ocular hypertension in the utilized mouse model, and transgenic deletion of *IκKβ* inhibited the NF-κB activity in astroglia.

### Effects of astroglial *IκKβ* deletion on astroglial inflammatory responses in experimental glaucoma

**Fig. 2:**
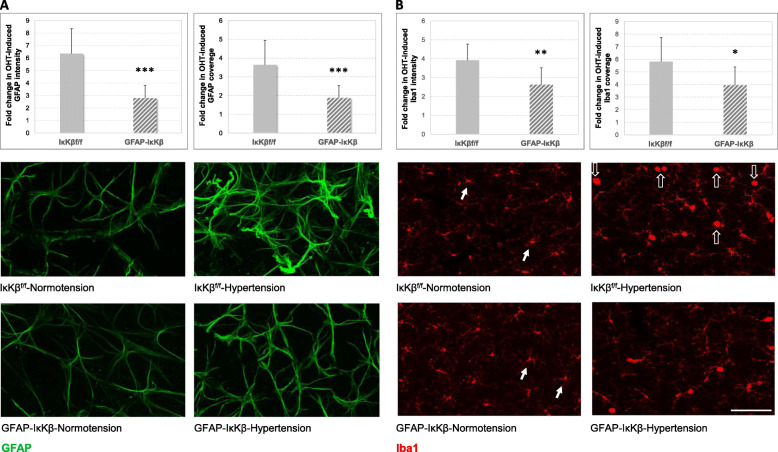
Effects of astroglial *IκKβ* deletion on glial morphological alterations in the ocular hypertensive mouse retina. Images in panel **a** present GFAP immunolabeling in retinal whole mounts from GFAP-IκKβ mice and controls (IκKβ^f/f^ wild-type for *cre*) with or without ocular hypertension (OHT). While the ocular hypertensive IκKβ^f/f^ retina presented reactive astrocytes exhibiting a hypertrophic morphology and prominent immunolabeling for GFAP (green), the ocular hypertension-induced GFAP immunolabeling was decreased in the retina with astroglial *IκKβ* deletion. Presented images were collected at the mid-periphery of retinal whole mounts. We analyzed the intensity (reflects the GFAP expression level of astroglia) and coverage (reflects the size of individual cells) of GFAP immunolabeling. As presented by bar graphs, both the intensity and the coverage of GFAP immunolabeling were less in ocular hypertensive GFAP-IκKβ retinas than ocular hypertensive IκKβ^f/f^ controls (***P < 0.001). As presented in panel **b**, based on Iba1 immunolabeling (red), the microglial response to ocular hypertension was also less obvious in GFAP-IκKβ retinas compared to transgenic controls. Microglia in normotensive retinas exhibited ramified morphology (white arrows), while reactive microglia with ocular hypertension displayed an ameboid morphology (translucent arrows). This morphological response was less prominent in ocular hypertensive GFAP-IκKβ retina relative to ocular hypertensive IκKβ^f/f^ control. As presented by bar graphs**,** both the intensity and the coverage of Iba1 immunolabeling were also less in ocular hypertensive GFAP-IκKβ retinas than ocular hypertensive IκKβ^f/f^ controls (**P = 0.002, *P = 0.02, respectively). Presented data (mean ± SD) represents a minimum of 11 mice per group (scale bar, 100 μm)

We next studied transgenic effects on astroglial responses to ocular hypertension by analyzing the quantitative measures of immunolabeling for GFAP. The astroglia labeled for this marker protein were distributed throughout the mouse retinal whole mounts. Initial hand-counting of GFAP+ cells did not detect a prominent difference in GFAP+ cell density between GFAP-IκKβ mice and IκKβ^f/f^ controls (80.00 ± 21.12 versus 81.50 ± 19.78 cells/mm^2^; *P* > 0.05). Similarly, the mean density of GFAP+ cells did not differ with the induction of ocular hypertension in these animals (82.00 ± 25.56 and 83.57 ± 28.95 cells/mm^2^ in GFAP-IκKβ and IκKβ^f/f^, respectively; *P* > 0.05). Although the overall density of GFAP+ cells remained unchanged, astroglial cells exhibited a hypertrophic morphology in ocular hypertensive eyes (relative to saline-injected normotensive controls). Based on image analysis, there was a widespread increase in both the intensity (reflecting the GFAP expression level of astroglia) and the coverage (reflecting the size of individual cells) of GFAP immunolabeling (Fig. 2a). The GFAP coverage may also reflect cell density; however, the GFAP+ cell counts did not reveal a prominent density variation as presented above. This increase was considered to indicate astroglial reactivity to ocular hypertension. Despite a prominent increase in the intensity and the coverage of GFAP immunolabeling with ocular hypertension, both of these imaging parameters were decreased (approximately 56% and 48%, respectively) in ocular hypertensive GFAP-IκKβ retinas relative to ocular hypertensive *IκKβ*^*f/f*^ controls. The glaucoma-related fold increase in intensity (*P* < 0.001) and coverage (*P* < 0.001) of the retinal GFAP immunolabeling were significantly lower in GFAP-IκKβ mice than IκKβ^f/f^ controls.

Interestingly, besides these transgenic effects on the ocular hypertension-induced astroglial GFAP response, some effects were also detectable on the immunolabeling pattern of reactive microglia. As shown in Fig. 2b, the morphological response of microglia to ocular hypertension (a shift from ramified morphology to reactive morphology) was less prominent in ocular hypertensive GFAP-IκKβ retinas than ocular hypertensive IκKβ^f/f^ controls. In addition, there was an increase in retinal Iba1 immunolabeling of microglia with ocular hypertension. However, microglial Iba1 immunolabeling was decreased in ocular hypertensive GFAP-IκKβ retinas relative to ocular hypertensive retinas from IκKβ^f/f^ mice. Both the intensity and the coverage of Iba1 immunolabeling were over 30% less in ocular hypertensive GFAP-IκKβ retinas than ocular hypertensive IκKβ^f/f^ controls (*P* = 0.002, *P* = 0.02, respectively).

**Fig. 3:**
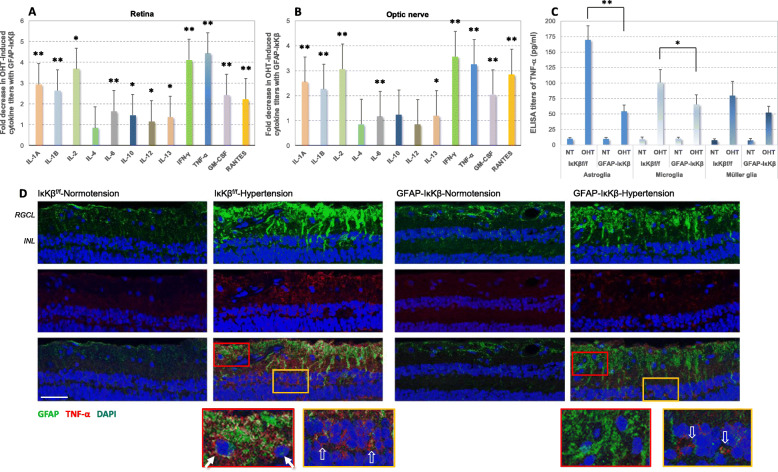
Effects of astroglial *IκKβ* deletion on neuroinflammatory responses of the ocular hypertensive mouse retina. In order to determine the inflammatory status of retina (**a**) and optic nerve (**b**) tissues, cytokine titers were analyzed by ELISA. We detected significantly reduced production of pro-inflammatory cytokines in ocular hypertensive GFAP-IκKβ eyes compared to ocular hypertensive controls (IκKβ^f/f^ mice wild-type for *cre*). Bar graphs show fold decrease in ocular hypertension (OHT)-induced pro-inflammatory cytokine production with GFAP-IκKβ. Data (mean ± SD) from retina and optic nerve samples are presented by separate graphs (represents a minimum of 4 mice per group; **P < 0.001, *P < 0.05). **c** Isolated samples of retinal astroglia and microglia (by immunomagnetic cell selection) also presented reduced titers of TNF-α (a major pro-inflammatory cytokine relevant to glaucomatous neurodegeneration) in ocular hypertensive GFAP-IκKβ than ocular hypertensive IκKβ^f/f^ controls. However, there was no significant difference between the TNF-α titers in normotensive (NT) samples from GFAP-IκKβ or IκKβ^f/f^ mice (*P* > 0.05). Reduced production of TNF-α with GFAP-IκKβ was more significant in astroglia (**P < 0.001) than microglia (*P = 0.02). When the isolated samples of retinal Müller glia were similarly analyzed, no significant difference was detectable in the ocular hypertension-induced TNF-α production of Müller glia between GFAP-IκKβ mice and IκKβ^f/f^ controls (*P* = 0.06). **d** Astroglial pro-inflammatory phenotype was also studied by immunohistochemical analysis. Presented are TNF-α immunolabeling of retinal tissue sections (scale bar, 100 μm), and red or yellow boxed areas are shown in higher magnification. TNF-α immunolabeling (red) of GFAP+ astroglia (green) was prominently higher in ocular hypertensive IκKβ^f/f^ retina (white arrows) than normotensive controls. However, astroglial TNF-α immunolabeling was not detectable in the RGC layer (corresponding to astrocytes), but still detectable in the inner nuclear layer (corresponding to Müller glia; translucent arrows) of ocular hypertensive GFAP-IκKβ retinas. Blue indicates nuclear DAPI staining. RGCL, and INL mark retinal ganglion cells layer, and inner nuclear layer, respectively

Since reactive astroglia produce increased amounts of pro-inflammatory cytokines, we also studied the pro-inflammatory phenotype by multi-cytokine assay of retina and optic nerve proteins. There was no detectable difference between the baseline cytokine titers in normotensive eyes of GFAP-IκKβ mice and IκKβ^f/f^ controls (*P* > 0.05). Similar to previous studies [25], ocular hypertension induced pro-inflammatory cytokine production in the retina and optic nerve of IκKβ^f/f^ control mice; however, pro-inflammatory cytokine titers were significantly lower in ocular hypertensive GFAP-IκKβ retinas and optic nerves. As presented in Fig. 3a and b, we detected more than three-fold lower enzyme-linked immunosorbent assay (ELISA) titers of pro-inflammatory cytokines in ocular hypertensive GFAP-IκKβ retinas and optic nerves compared to ocular hypertensive IκKβ^f/f^ controls. Among the studied cytokines that are known to be transcriptional targets for NF-κB, interleukins (IL), including IL-1A (*P* < 0.001), IL-1B (*P* < 0.001), IL-2 (*P* = 0.01), IL-6 (*P* < 0.001), IL-10 (*P* = 0.03), IL-12 (*P* = 0.04), IL-13 (*P* = 0.02), interferon-gamma (IFN-γ) (*P* < 0.001), and TNF-α (*P* < 0.001) exhibited significantly lower titers in ocular hypertensive retinas of GFAP-IκKβ mice compared to ocular hypertensive IκKβ^f/f^ controls. Similar to retina, optic nerve titers of IL-1A (*P* < 0.001), IL-1B (*P* < 0.001), IL-2 (*P* < 0.001), IL-6 (*P* < 0.001), IL-13 (*P* = 0.046), IFN-γ (*P* < 0.001), and TNF-α (*P* < 0.001) were significantly lower in ocular hypertensive GFAP-IκKβ eyes than ocular hypertensive IκKβ^f/f^ controls. Note that these data reflecting the whole tissue provide information about the overall pro-inflammatory status of the retina and optic nerve.

We also analyzed the isolated samples of retinal astroglia and microglia for TNF-α that is a major pro-inflammatory cytokine relevant to glaucomatous neurodegeneration [6, 9, 32], also a transcriptional target for NF-κB [33, 34]. There was no significant difference between the TNF-α titers in normotensive samples from GFAP-IκKβ or IκKβ^f/f^ mice (*P* > 0.05); however, ocular hypertension-induced production of this cytokine was significantly lower in ocular hypertensive GFAP-IκKβ than ocular hypertensive IκKβ^f/f^ controls. As presented in Fig. 3c, reduced production of TNF-α with GFAP-IκKβ was more significant in astroglia (*P* < 0.001) than microglia (*P* = 0.02). When we similarly analyzed TNF-α titers in isolated samples of retinal Müller glia, no statistically significant decrease was detectable in the ocular hypertension-induced production of TNF-α in GFAP-IκKβ mice compared to ocular hypertensive IκKβ^f/f^ controls (*P* = 0.06).

TNF-α production was also studied by immunohistochemistry for further validation. Figure 3d shows GFAP and TNF-α double immunolabeling of ocular hypertensive GFAP-IκKβ and IκKβ^f/f^ retinas. Tissue immunolabeling supported increased production of this pro-inflammatory cytokine in GFAP-labeled astroglia in ocular hypertensive eyes; however, the ocular hypertension-induced TNF-α immunolabeling was prominently decreased in GFAP-IκKβ retinas compared to ocular hypertensive IκKβ^f/f^ controls. TNF-α immunolabeling of the GFAP+ astroglia was not detectable in the RGC layer (corresponding to astrocytes), but still detectable in the inner nuclear layer (corresponding to Müller glia) of the ocular hypertensive GFAP-IκKβ retina.

Thus, GFAP-labeled astroglia survived *IκKβ* deletion over the 12 weeks of experimental period with ocular hypertension and exhibited decreased inflammatory activity as exemplified by decreased production of pro-inflammatory/pro-apoptotic cytokines, including TNF-α.

### Effects of astroglial *IκKβ* deletion on neurodegenerative outcomes of experimental glaucoma

**Fig. 4:**
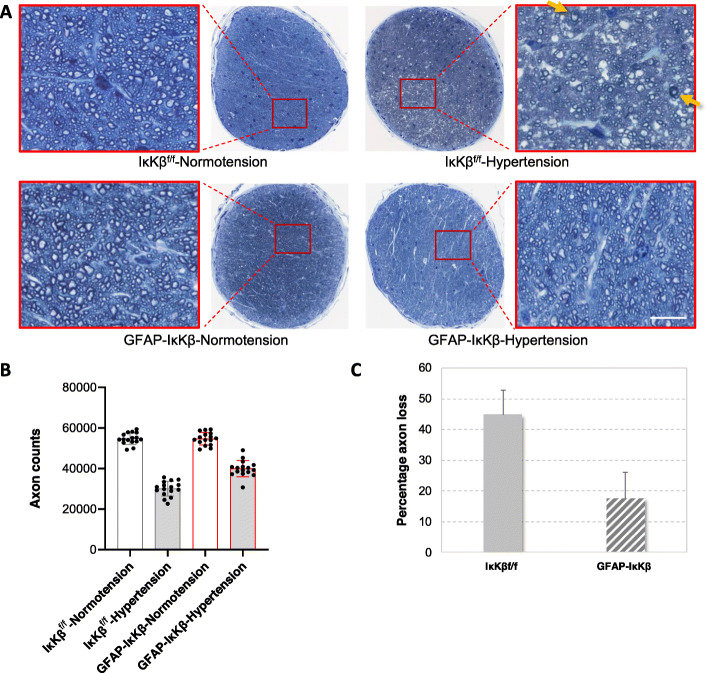
Transgenic effects on optic nerve axon counts. Panel **a** presents composite images of optic nerve cross-sections stained with 2% toluidine blue. Red boxed areas are shown in higher magnification (scale bar, 100 μm). Optic nerve axons were counted in these cross-sections, and the neuron loss was determined in ocular hypertensive eyes after adjusting to normotensive fellow eyes. Note the prominent axon loss and gliosis in ocular hypertensive control eye (IκKβ^f/f^ mice wild-type for *cre*). Yellow arrows show degenerating axons and myelin debris. However, optic nerve structure was well preserved in ocular hypertensive GFAP-IκKβ, and the number of remaining axons was significantly higher than ocular hypertensive IκKβ^f/f^ control (***P < 0.001). Bar graphs in panel **b** present axon counts in GFAP-IκKβ mice and IκKβ^f/f^ controls with or without experimentally induced ocular hypertension, and bar graphs in panel **c** show percentage of axon loss in ocular hypertensive transgenic or control animals. Presented data (mean ± SD) represents a minimum of 16 mice per group. Transgenic deletion of astroglial *IκKβ* resulted in an approximately 60% protection of axons in ocular hypertensive eyes

Our following analyses aimed to determine the effects of astroglial NF-κβ inhibition on neurodegenerative outcomes of experimental glaucoma. We therefore comparatively analyzed RGC and axon counts in GFAP-IκKβ and control eyes with or without ocular hypertension. Figure 4 shows optic nerve cross-sections, and the accompanying graphs present the number of optic nerve axons and the rate of axon injury over the experimental period. When we counted neurons, we detected a significant decrease in the number of remaining axons in ocular hypertensive eyes relative to normotensive controls (*P* < 0.001). After adjusting to normotensive fellow eyes, the axon counts indicated approximately 45% loss with 12 week of ocular hypertension in IκKβ^f/f^ controls, but 18% loss in GFAP-IκKβ mice. Thus, despite similar elevation of intraocular pressure, the axon loss was approximately 60% less in ocular hypertensive GFAP-IκKβ than ocular hypertensive controls. Besides a significant reduction in axon counts, microscopy of optic nerve cross-sections from ocular hypertensive eyes demonstrated swollen and degenerating axons, myelin debris, and gliotic scar. However, compared to ocular hypertensive IκKβ^f/f^ mice, ocular hypertensive GFAP-IκKβ mice exhibited a preserved morphology of optic nerve axons with fewer degenerating profiles.

**Fig. 5:**
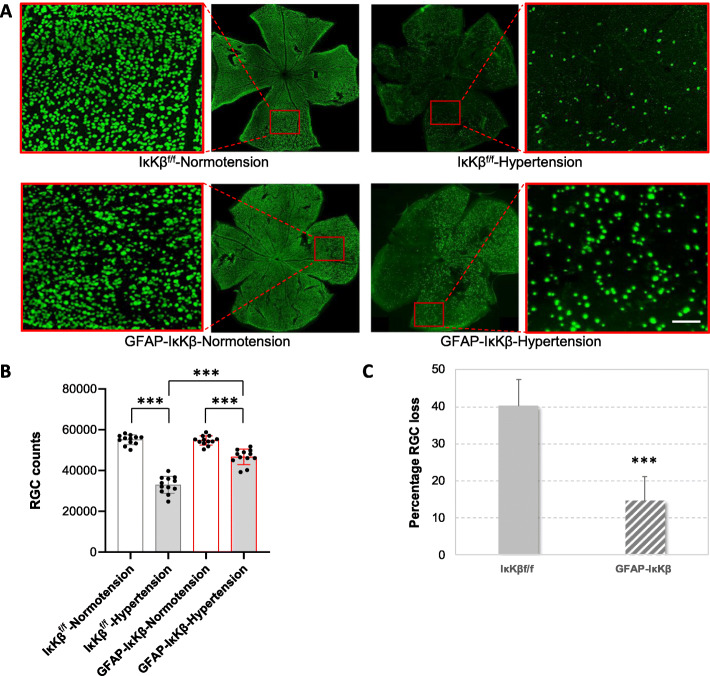
Transgenic effects on RGC counts. **a** Composite images of the whole-mounted retinas immunolabeled for RPBMS, an RGC marker. Red boxed areas are shown in higher magnification (scale bar, 100 μm). Ocular hypertensive control eyes (IκKβ^f/f^ mice wild-type for *cre*) exhibited a visible loss of RPBMS-labeled RGC somas compared to normotensives; however, RGC bodies were well protected in ocular hypertensive eyes of GFAP-IκKβ mice. RPBMS-labeled RGC somas were counted and the neuron loss was determined in ocular hypertensive eyes after adjusting to normotensive fellow eyes. The number of RPBMS-labeled RGCs was significantly higher in ocular hypertensive GFAP-IκKβ retinas than ocular hypertensive IκKβ^f/f^ controls (***P < 0.001). Bar graphs in panel **b** present RGC counts in GFAP-IκKβ mice and IκKβ^f/f^ controls with or without experimentally induced ocular hypertension, and bar graphs in panel **c** show percentage of RGC loss in ocular hypertensive transgenic or control animals. Presented data (mean ± SD) represents a minimum of 16 mice per group. Transgenic deletion of astroglial *IκKβ* resulted in an approximately 63% protection of RGC somas in ocular hypertensive eyes

Neuron injury was also analyzed in retinal whole mounts after immunolabeling of RGCs for a marker protein, RPBMS. As presented in Fig. 5, this analysis demonstrated RPBMS-labeled RGC bodies spanning the entire area in normotensive eyes. Similar to optic nerve axons, a significant decrease was detectable in the number of RGCs with ocular hypertension. However, despite visible loss of RPBMS-labeled RGCs in ocular hypertensive eyes of IκKβ^f/f^ controls, ocular hypertensive GFAP-IκKβ retinas exhibited a greater number of RGCs (*P* < 0.001). The RGC counts in ocular hypertensive eyes (when adjusted to normotensive fellow eyes) were approximately 40% at 12 weeks of ocular hypertension in controls, but 15% in GFAP-IκKβ mice. Thus, similar to the transgenic effect detected on optic nerve axon loss, GFAP-IκKβ provided approximately 63% protection to RGC somas.

**Fig. 6:**
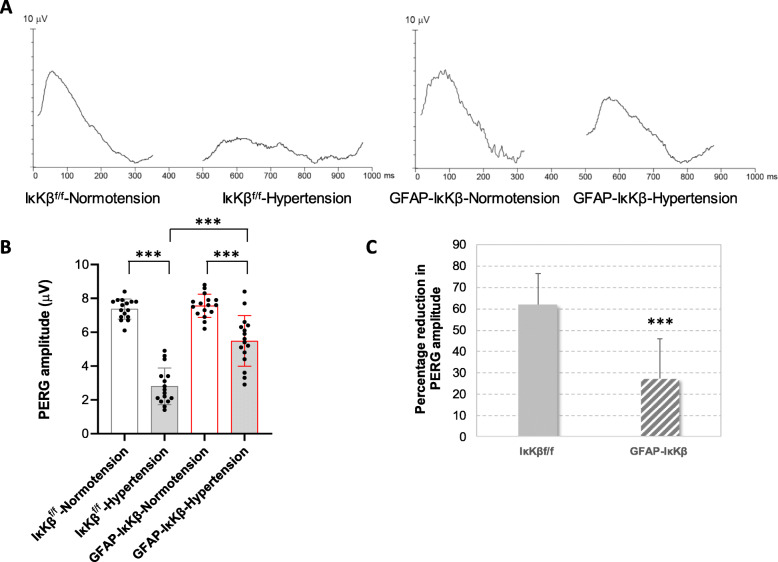
Transgenic effects on pattern electroretinography (PERG) responses. Panel **a** shows PERG responses in GFAP-IκKβ mice and controls (IκKβ^f/f^ mice wild-type for *cre*) with or without experimentally induced ocular hypertension. Bar graphs in panel **b** present PERG amplitude and bar graphs in panel **c** show percentage reduction in PERG amplitude in these transgenic or control animals. Presented data (mean ± SD) represents a minimum of 16 mice per group. Transgenic deletion of astroglial *IκKβ* resulted in preserved PERG amplitude in ocular hypertensive eyes (***P < 0.001)

In addition to neuron counts, we also analyzed RGC function by recording the PERG responses (Fig. 6). Ocular hypertensive eyes exhibited an impaired neuron function as assessed by reduced PERG amplitude (*P* < 0.001). However, despite a prominent reduction of PERG amplitude after 12 weeks of ocular hypertension in IκKβ^f/f^ mice, it was preserved in ocular hypertensive eyes of GFAP-IκKβ mice. This observation supported that besides structural protection, astroglial NF-κβ inhibition protected PERG responses that are typically lost during the course of glaucomatous neurodegeneration in ocular hypertensive mouse eyes [27, 28].

Thus, transgenic inhibition of NF-κβ in astroglia improved the outcome measures of experimental glaucoma, including the inflammatory phenotype of glia, neuron (including both RGC axons and somas) counts, and PERG responses.

## Discussion

Using an astroglia-targeted conditional transgenic mouse model, this study indicated a key role for astroglial NF-κB in neuroinflammatory and neurodegenerative outcomes of experimental glaucoma. Based on the findings of this study, inhibition of astroglial NF-κB, a major transcriptional activator of neuroinflammation, can prevent neurodegenerative inflammation and increase neuron survival and function during glaucomatous neurodegeneration.

After conditional deletion of astroglial *IκKβ*, we detected a significant decrease in ocular hypertension-induced morphological and pro-inflammatory responses of NF-κB-inhibited astroglia in the retina and optic nerve. NF-κB is widely expressed in the brain, and its activity in different cell types presents a wide array of biological functions with cell-autonomous and non-cell-autonomous impacts [10]. Unlike neurons [35], glial cells only present slight NF-κB activity under basal conditions, which is implicated in maintaining brain homeostasis. However, excessive activation of NF-κB in reactive glia has been shown to be neurotoxic in chronic neurodegenerative diseases [10]. Evidently, activation of glial NF-κB and consequent production of inflammatory mediators can exacerbate neuronal cell death, and its inhibition reduces disease severity after brain injury [36, 37]. Similar to neurons, the predominantly activated form of NF-κB in reactive astrocytes contains the RelA/p65 subunit, rather than the RelB/p52 heterodimers that mediate non-canonical signaling. Our experimental strategy targeted *IκKβ* that stimulates the nuclear import of the RelA/p65 subunit for the transcriptional activity of NF-κB [30, 31]. We found that ocular hypertension-induced p65 phosphorylation was inhibited in *IκKβ*-deleted astroglia, and the inhibition of astroglial NF-κB impeded inflammatory and neurodegenerative outcomes of ocular hypertension-induced experimental glaucoma in mice.

Our findings indicated that the inhibition of astroglia-driven neuroinflammation can protect both axons and somas of RGCs in experimental glaucoma. Indeed, besides the RGC soma injury evident in glaucoma, glial NF-κB-mediated inflammation has been implicated in the self-destruction of injured axons [13, 38], which is relevant to glaucomatous axon pathology [5]. Evidently, NF-κB-regulated pro-inflammatory cytokines, such as TNF-α [9, 39] or FasL [40] secreted by reactive astrocytes, can induce RGC apoptosis and axon degeneration. Additionally, the TNF-α secreted by glial cells may induce the death of oligodendrocytes [41] that constitute a critical cell type for axon health by providing metabolic support [42], as well as producing myelin. Moreover, astroglial NF-κB has been linked to dendrite degeneration and synapse dysfunction through complement-mediated processes in neurodegenerative diseases [43].

Besides the protection of neuron structure, our observations through PERG recordings were also supportive of a functional protection against experimental glaucoma in GFAP-IκKβ mice. The PERG responses have been proven to be an important tool to detect and monitor RGC dysfunction in human glaucoma; they also exhibit dramatic worsening in mouse glaucoma [27, 28] and can detect a treatment effect in mice [44, 45]. The severe decrease that we detected in the PERG amplitude reflected glaucoma-related injury to RGC synapses, dendrites, or soma. However, protected PERG function after NF-κB inhibition in astroglia was supportive of reduced inflammatory/neurodegenerative outcomes. Interestingly, some previous evidence also suggests that astroglial NF-κB may directly participate in the regulation of synaptic plasticity in brain neurons [46]. Thus, with respect to the widespread nature of astroglia-driven inflammatory processes during glaucomatous neurodegeneration, immunomodulation offers a treatment strategy to provide protection against inflammatory injury to RGCs at different neuronal compartments. Astroglial NF-κB appears to be a critical target to achieve this aim.

A different mouse model with an overexpression of a dominant negative form of *IκBα* in astroglia has been studied in other injury models of the optic nerve and has improved neuron survival. For example, inhibition of astroglial NF-κB has suppressed chronic inflammation in experimental autoimmune encephalomyelitis and has protected against optic nerve damage and RGC loss in this optic neuritis model for multiple sclerosis [11, 13]. In addition, the inactivation of astroglial NF-κB has reduced pro-inflammatory genes and has promoted RGC survival after retinal ischemia [12]. The findings of the current study using a conditional transgenic model for IκKβ deletion in astroglia provided evidence for the immunomodulatory and neuroprotective potential of astroglial NF-κB inhibition in experimental glaucoma.

Based on the findings of this study, targeting the astroglial NF-κB appears to be a logical strategy for immunomodulation to avoid inflammatory injury to neurons in glaucoma, which is also endorsed by the accumulated data in the field. Growing datasets generated by transcriptomic or proteomic profiling have unveiled the early upregulation of numerous molecules linked to inflammation pathways in the retina and optic nerve of human donor eyes or animal models with glaucoma. The NF-κB activation pathway was among the molecular pathways most affected in glaucomatous astroglia in these studies [6, 7]. Importantly, many of the identified inflammation pathways in glaucomatous glia, including TNF-α/TNFR and TLR signaling, inflammasome activation, and complement dysregulation, are commonly related to a NF-κB-regulated transcriptional program [6–8]. The inflammatory outcomes of oxidative stress during glaucomatous neurodegeneration [47] and the immunomodulatory potential of antioxidant treatment [25] also link this redox-sensitive transcription factor in glia-driven neuroinflammation in glaucoma.

Inhibition of astroglial NF-κB by GFAP-IκKβ resulted in a prominent decrease in astroglial TNF-α, a transcriptional target of NF-κB [33, 34]. This observation signifies astroglial NF-κB as a treatment target to inhibit the neurodegenerative outcomes of TNF-α signaling in glaucoma. Based on previous studies of glaucoma, TNF-α presents increased astroglial production in the glaucomatous human retina [32] and optic nerve [48]. In addition, this major pro-inflammatory and neurotoxic cytokine participates in neuroinflammatory outcomes [6, 7, 49], RGC apoptosis, oligodendrocyte death, and axon loss during glaucomatous neurodegeneration [9, 39, 41].

Numerous other pro-inflammatory cytokines (and chemokines) known to be NF-κB’s target genes, such as IFN-γ, IL-1, IL-2, IL-12, IL17 [50–53], or iNOS [54], also exhibit astroglial upregulation during neurodegeneration in glaucoma. Moreover, various extracellular matrix molecules that are gene targets of NF-κB (such as tenascin-C that is upregulated in glaucomatous astroglia [55]) are important components of astrogliosis. By promoting tissue stiffening and activating degenerative signaling, astrogliosis may also contribute to axon injury and dendrite pathology in glaucoma [4]. Also notable, interactions of cytokines and chemokines with adhesion molecules (also regulated by NF-κB) on the vascular endothelium and astrocyte end-feet may be involved in the trafficking of circulating immune cells across the blood-brain barrier in glaucomatous tissues [56–58]. In addition to NF-κB-regulated immunoreceptors (such as MHC molecules [59] or RAGE [60] that are upregulated on glaucomatous astroglia), other NF-κB targets known to be involved in antigen presentation may also play a critical role in astroglia-mediated activation of systemic immunity in glaucoma [1, 4]. Hence, besides the pro-inflammatory cytokine response presented herein, transgenic inhibition of other NF-κB-regulated processes might also be involved in the immunomodulatory and neuroprotective outcomes detected in this study. Another interesting aspect that our ongoing work pursues is whether therapeutic inhibition of astroglial inflammatory activity may lessen the increased energy need of astroglia and recover astroglial metabolic support to RGCs.

The findings of this study encourage immunomodulation strategies targeting the NF-κB in astroglia; however, it is important to emphasize that the complexity of cell type-specific (and subunit-specific) roles of NF-κB makes it a challenging treatment target. Although NF-κB plays an important role in glia-driven neuroinflammation and secondary injury processes, this inducible transcription factor also activates critical anti-apoptotic genes [61, 62] and regulates a broad range of processes essential for neuron survival, synapse formation, and plasticity [63, 64]. This is valid for RGCs as well, which progressively die in NF-κB1 knockouts [65]. Hundreds of reported inhibitors of NF-κB, including small molecule inhibitors, lack the cell type specificity, and thus interfere with NF-κB’s physiological roles, including those in neuron survival. Therefore, targeted manipulation, specifically of astroglial NF-κB within the eye, seems ideal to prevent or reverse neuroinflammation in glaucoma while keeping the systemic immune defense intact with no risk of undesired side effects on neurons. We hope that the developing gene engineering techniques and new delivery tools, such as astroglia-specific viral vectors [66], will quickly enable the direct translation of experimental information into clinical manipulation of neurotoxic inflammation for glaucoma treatment, in a cell type-targeted manner.

Continued efforts on potential astroglia-targeting treatments should also explore additional aspects. For example, the degeneration of RGCs in glaucoma is asynchronized over a chronic disease period, through which astroglia exhibit a persisting reactive phenotype with multiple activation states. It should be clarified whether the inhibition of astroglial NF-κB may affect the plasticity or neurosupportive functions of these glia over a long period; how the timing or duration of an astroglia-targeting treatment may be optimized; and whether a combination with other treatments (such as lowering elevated intraocular pressure) may further improve the outcome.

In addition to the decreased inflammatory activity of astroglia after *IκKβ* deletion, we also detected a decrease in the ocular hypertension-related microglial response in GFAP-IκKβ retinas. This interesting observation may support the importance of astroglia-derived factors to shape microglia responses. Indeed, non-cell-autonomous functions of astroglial NF-κB have been shown to include the regulation of microglial responses in neurodegenerative disease models [67]. Notably, astroglia and microglia perform complementary and synchronic roles during neuroinflammation. Besides serving as primary immune cells, these glia closely interplay to regulate each other’s phenotype and immune functions [68]. Cytokines are involved in the regulation of direct molecular conversation between the glial cell types. While astroglial cytokines and chemokines chemoattract and activate microglia, the activated microglia induce neurotoxic A1 astrocytes by secreting cytokines, such as IL-1α, and TNF-α [69]. NF-κB, the key transcriptional activator of these pro-inflammatory molecules [34], is also activated in neurotoxic A1 astrocytes [43]. These imply the importance of NF-κB for the regulation of paracrine and autocrine feedback loops between astroglia and microglia in neuroinflammation. However, besides their direct communication, astroglia and microglia also respond to shared environmental signals, including the signals arising from stressed/injured RGCs [4]. In light of current knowledge, we question whether decreased microglial response to ocular hypertension in GFAP-IκKβ mice might be related to a NF-κB-regulated cross-talk between astroglia and microglia during neuroinflammation and neurodegeneration, and/or might be secondary to decreased neuron injury in these transgenic animals. Thus, although this study was focused on GFAP-expressing astroglia, further elucidation of the microglial response and astroglia-microglia interactions in glaucomatous neuroinflammation and neurodegeneration is also of high interest. Ongoing studies are expected to expand the present information through a comparative analysis of glial subtype-targeted (astroglia-targeted and microglia-targeted) models. Further studies to distinguish between resident microglia versus infiltrating macrophages are also highly appealing.

Another important point to note for glial subtype responses is related to the GFAP-cre/ERT2 [20] that carries a cre transgene under the control of the GFAP promoter that we used for generating our astroglia-targeted mouse line. The mouse lines with GFAP-targeted cre expression, which present more than 95% recombination efficiency and specificity [70], have been used as a safe and reliable method for the transgenic deletion of targeted genes. When considering the conditional GFAP-cre/ERT2 line utilized in this study, neither the cre expression nor the tamoxifen treatment caused detectable changes in retinal structure, RGC function, neuronal vulnerability, or glial reactivity in the mouse retina [71]. Similarly, we did not detect any alterations in GFAP expression, glial morphology, or cytokine production by cre or tamoxifen exposure alone. However, ocular hypertension-induced glial inflammatory responses exhibited a prominent reduction by tamoxifen-induced cre/lox-based deletion of *IκKβ* in GFAP-expressing astroglia.

Although GFAP can provide astrocyte-specific information when focused on the optic nerve, it is important to highlight that this commonly utilized marker may also be expressed by Müller glia (as well as astrocytes) in the retina, particularly in glaucoma. However, no prominent cre-recombinase immunolabeling was detectable in Müller glia, and their phospho-p65 immunolabeling persisted in GFAP-IκKβ mice. In support of this observation, analysis of isolated Müller glia samples and the immunolabeling of retinas did not detect a significant decrease in the ocular hypertension-induced TNF-α production of Müller glia in GFAP-IκKβ mice. We wonder whether these observations in Müller glia may be related to a lower expression level of GFAP (compared to retinal astrocytes) and/or a possibility of non-canonical NF-κB signaling in these glia. In order to further dissect the NF-κB-regulated responses of astrocytes and Müller glia in the glaucomatous retina, our work continues to comparatively analyze isolated samples of Müller glia from GFAP-IκKβ mice and a preferentially Müller glia-targeted line. Since the possibility of IκKβ deletion in Müller glia (as well as in astrocytes) by GFAP-IκKβ cannot be fully ruled out, we consistently used the term “astroglia” (not astrocytes), as this term also covers Müller glia in the retina. An alternative term could be macroglia; however, since other macroglial cells (including oligodendrocytes and lamina cribrosa cells, both relevant to glaucomatous neurodegeneration) do not express GFAP, we considered astroglia as the most appropriate term.

In summary, the findings of this study establish a causative relationship between astroglial NF-κB and neuroinflammatory/neurodegenerative outcomes of experimental glaucoma and value this axis as a favorable treatment target for immunomodulation and neuroprotection. In addition to upcoming treatments that target the neuron injury itself (and current treatments that target elevated intraocular pressure, a major risk factor), the targeting of glia-mediated secondary injury processes seems to be very important in clinical setting. This is because patients with glaucoma are usually diagnosed at advanced stages when even the control of ocular hypertension by treatment cannot prevent the secondary component of disease progression. Neurodegeneration may continue through a vicious cycle of prolonged tissue stress and injury, glial responses, sustained release of neurotoxic inflammatory mediators, and dysregulation of immune homeostasis.

## Conclusions

Glia-driven neuroinflammation has increasingly been recognized to play a critical role in glaucomatous neurodegeneration, and its modulation promises for neuroprotection. Through a cell type-specific experimental approach using an astroglia-targeted conditional transgenic mouse model, this study demonstrated an important role for astroglial NF-κB in neuroinflammatory and neurodegenerative outcomes of experimental glaucoma. The findings of this study also supported the potential of astroglial NF-κB as an immunomodulation target to increase neuron survival and function in this blinding disease. By pointing to a potential treatment strategy targeting the astroglia, which can eliminate the risk of undesired side effects on neurons, these experimental findings promote a translational path for improved treatment of glaucoma and other inflammatory neurodegenerative diseases.

## Data Availability

Data and materials are available to interested parties upon request.

## Abbreviations

ACSA-2: Astrocyte cell surface antigen-2
GFAP: Glial fibrillary acidic protein
IκKβ: Inhibitor of kappaB kinase-subunit beta
IFN-γ: Interferon-gamma
IL: Interleukin
NF-κB: Nuclear factor-kappaB
PERG: Pattern electroretinography
RGC: Retinal ganglion cell
RPBMS: RNA-binding protein with multiple splicing
TLR: Toll-like receptor
TNF-α: Tumor necrosis factor-alpha
TNFR: Tumor necrosis factor receptor
